# KGRDR: a deep learning model based on knowledge graph and graph regularized integration for drug repositioning

**DOI:** 10.3389/fphar.2025.1525029

**Published:** 2025-02-11

**Authors:** Huimin Luo, Hui Yang, Ge Zhang, Jianlin Wang, Junwei Luo, Chaokun Yan

**Affiliations:** ^1^ School of Computer and Information Engineering, Henan University, Kaifeng, China; ^2^ Henan Key Laboratory of Big Data Analysis and Processing, Henan University, Kaifeng, China; ^3^ College of Computer Science and Technology, Henan Polytechnic University, Jiaozuo, China; ^4^ Academy for Advanced Interdisciplinary Studies, Henan University, Zhengzhou, China

**Keywords:** drug repositioning, drug-disease interaction prediction, multi-similarity fusion, biomedical knowledge graph, feature fusion

## Abstract

Computational drug repositioning, serving as an effective alternative to traditional drug discovery plays a key role in optimizing drug development. This approach can accelerate the development of new therapeutic options while reducing costs and mitigating risks. In this study, we propose a novel deep learning-based framework KGRDR containing multi-similarity integration and knowledge graph learning to predict potential drug-disease interactions. Specifically, a graph regularized approach is applied to integrate multiple drug and disease similarity information, which can effectively eliminate noise data and obtain integrated similarity features of drugs and diseases. Then, topological feature representations of drugs and diseases are learned from constructed biomedical knowledge graphs (KGs) which encompasses known drug-related and disease-related interactions. Next, the similarity features and topological features are fused by utilizing an attention-based feature fusion method. Finally, drug-disease associations are predicted using the graph convolutional network. Experimental results demonstrate that KGRDR achieves better performance when compared with the state-of-the-art drug-disease prediction methods. Moreover, case study results further validate the effectiveness of KGRDR in predicting novel drug-disease interactions.

## 1 Introduction

The traditional drug development (R&D) process is extremely expensive, lengthy, complex, and risky (Chong and Sullivan, 2007). According to a recent study (Chan et al., 2019), introducing a new drug to the market involves multiple steps, typically costs over 2 billion USD, and takes an average of 12 years. Effectively improving the success rate of R&D and reducing the expensive workload of the verification procedure has become an urgent challenge for researchers (Wang et al., 2023). Drug repositioning is an approach to finding new therapeutic potential for existing drugs that have already been approved by the Food and Drug Administration (FDA) for the treatment of diseases (Novac, 2013). This innovative strategy has many advantages, such as reducing drug risk, shortening clinical evaluation cycle, enhancing cost-effectiveness, and improving efficiency (Pushpakom et al., 2019; Luo et al., 2021). In recent years, drug repositioning has been widely applied in disease and related therapeutic areas, including anticancer drug discovery (Ye et al., 2014), identification of novel therapies for orphan and rare diseases (Setoain et al., 2015), overcoming of drug resistance (Younis et al., 2015) and advancement of personalized medicine (Li and Jones, 2012). These successful applications have shown that drug repositioning is increasingly becoming an attractive proposition (Lotfi Shahreza et al., 2018).

Many previous studies of computational drug repositioning mainly utlized drug and disease similarity information to predict potential drug-disease associations (Wang et al., 2019; Zhang et al., 2018; Luo et al., 2018). However, most of these methods typically used a single type of similarity information, which can lead to various data quality issues, such as missing information, sparse data and insufficient generalization ability. Based on various biomedical information, multiple drug similarities and disease similarities can be calculated (Huang et al., 2021). These similarities can be integrated to enhance the feature representation of drugs and diseases in multiple dimensions (Peng et al., 2021). In recent years, many methods have been proposed to integrate multiple similarity information to improve the performance of drug-disease interaction prediction. These integration methods primarily use two main schemes including linear-based and nonlinear-based strategies to handle multiple similarities.

The similarity integration methods based on the first strategy train the prediction model by jointly learning linear combination of multiple similarities. AVE (Nascimento et al., 2016) was the most intuitive linear approach that simply averaged multiple similarity networks by assigning the same weight to each network. Hilbert–Schmidt Independence Criterion (HSIC) (Ding et al., 2020) applied the HSIC metric to achieve the optimal combination of different similarity networks. This method utilized multi-kernel learning to assign weights to each similarity network, thereby maximizing the dependency on the ideal similarity network. Local Interaction Consistency (LIC) (Liu and Tsoumakas, 2021) introduced the concept of local balance, which refers to the proportion of similar drugs or diseases with the same drug-disease interaction. This method improves the prediction performance by assigning higher weights to similarity networks with better local balance. Liu et al. (2023) proposed a fine-grained selective similarity integration method (FGS), which further used a similarity selection step based on LIC to filter out noise information with finer granularity. However, these linear-based similarity integration methods can’t capture complex relations among these networks effectively.

Integration methods using the second strategy regard each similarity network as a graph and exploit the structure of the graph to find complex nonlinear relations between network nodes. These integration methods can be divided into two categories: (1) methods using SNF (Wang et al., 2014), and (2) methods using matrix joint decomposition strategies. SNF used a nonlinear approach based on message-passing theory and updated each similarity network iteratively to make it more similar to the others, eventually converging to a single network. Recently several works have extended SNF in different ways to propose novel integration approaches. For example, considering that the Euclidean distance metric used in SNF suffers from the curse of dimensionality (Rozza et al., 2012), HSNF (Hierarchical SNF) (Liu and Shang, 2018) designed a hierarchical processing by applying the SNF method to different feature subsets multiple times. This method aims to reduce the noise and redundant information of high-dimensional data, thereby improving the quality of the fused similarity network. Although HSNF performs better than SNF on multiple datasets, it has a higher computational cost due to the iteration of SNF. Affinity Network Fusion (ANF) (Ma and Zhang, 2017) used affinity matrices to represent the degree of association between networks from different data sources and reduced the computational cost of SNF by simplifying the iterative integration process into a more straightforward one-step random walk approach. Considering the redundancy and noise problems in multi-similarity networks, several methods have been proposed to improve SNF. For example, similarity selection step is adopted to remove network noise in several integration methods (Olayan et al., 2018; Thafar et al., 2020). The Similarity Kernel Fusion algorithm (SKF) (Jiang et al., 2019) used the kernel functions to construct the kernel matrix of each similarity network and adjusted its weight according to the contribution of each kernel matrix to the target task, thereby increasing the weight of the similarity kernel with lower noise and improving the model’s performance. The association-signal-annotation boosted similarity network fusion (ab-SNF) method (Ruan et al., 2019) introduced the concept of associated signals and aimed to improve SNF by using a weighted distance measurement to emphasize important signal features while minimizing the impact of noisy data. The weight was measured using the paired *t*-test method, which calculates the weight ratio by comparing the negative sample with the adjacent normal sample at the feature.

Some studies employed joint matrix decomposition to differentiate shared information from network-specific information across various datasets and identify the consistency of multiple networks (Žitnik et al., 2015; Žitnik et al., 2013; Zheng et al., 2013). Recently, Cho et al. proposed a method for multi-similarity networks integration, named Mashup (MU) (Cho et al., 2015; 2016; Wang et al., 2015). This method combines random walk with multi-view factorization and provides a fruitful integration framework. Zhang et al. (2022b) developed a multi-similarity integration method, EnMUGR, that incorporates graph regularization. EnMUGR can effectively address noise and redundancy in multi-similarity networks.

In addition to using similarity information as feature representations of drugs and diseases, drug repositioning methods can also use the associations between drugs and other related biomedical entities (such as genes, diseases, and pathways) to learn feature representations, thereby more accurately predicting potential drug-disease association information (Zhang et al., 2023; Zhang et al., 2024). By constructing a knowledge graph that contains drugs and other related entities, feature representations of drugs and diseases can be learned, which include the relationships and contextual information between these entities. Domingo-Fernández et al. (2022) proposed a knowledge graph causal reasoning model (RPath) for drug discovery, which uses drug perturbation and disease-specific transcriptome features to help identify potential drug candidates for specific diseases by reasoning on causal paths in a knowledge graph (KG). Zhang et al. (2022a) combined drug chemical structures and biomedical knowledge graphs (KGs) to propose a meta-path-based graph representation learning model for drug-disease association (DDA) prediction, namely, RLFDDA. This model constructs a heterogeneous network by integrating DDA, disease-protein associations, and drug-protein associations, and adopts a meta-path random walk strategy to learn the latent representations of drugs and diseases. Han et al. (2023) proposed a multi-channel feature fusion model for multi-typed DDIs prediction, which employs a multi-channel feature fusion module to fuse drug chemical structure features, drug pairs’ extra label features, and KG features of drugs. This approach effectively alleviates the problem of feature redundancy and noise from KG.

In light of the above discussion, we proposed a novel computational framework for drug repositioning based on graph regularized integration and knowledge graph embedding. First, in order to address the problem of incomplete entity information caused by using only one source of data, we employ a graph regularized integration method with a denoised diffusion module to fuse multiple similarity information of drugs and diseases, thereby calculating the common attribute feature representation of drugs and diseases. Then we utilize knowledge graph embedding methods to learn global topological feature representations of drug and disease entities. Finally, we fuse these two learned feature representations by iAFF and feed them into a graph convolutional network prediction model to identify potential therapeutic indications for drugs.

The major contributions of this study are summarized as follows.

• This study proposed a novel framework KGRDR, which integrates a fusion algorithm based on graph regularization and knowledge graph embedding to identify potential indications for existing drugs, providing valuable insights to promote drug repositioning.
• The KGRDR framework utilized a graph regularized method to effectively address noise and redundancy in multi-similarity networks.
• An iterative attention feature fusion method is utilized to combine similarity feature information with the structural feature information derived from knowledge graph learning.

## 2 Materials and methods

### 2.1 Notations and brief review of KGRDR

Similarity matrices, denoted by ${\left\{S^{\left(u\right)}\right\}}_{u=1}^{u}$ , are calculated based on various biomedical data sources for drug pairs or disease pairs. $S_{r}^{\left(u\right)}\in R^{N_{r}\times N_{r}}$ are drug pairs similarity matrices, where $N_{r}$ is the number of drug nodes; $S_{d}^{\left(u\right)}\in R^{N_{d}\times N_{d}}$ are disease pairs similarity matrices, where $N_{d}$ is the number of disease nodes; element $s_{ij}^{\left(u\right)}$ represent the similarity score between nodes i and j in the $u^{th}$ network; ${\left\{S^{\left(v\right)}\right\}}_{v=1}^{v}$ denotes the similarity matrices selected from ${\left\{S^{\left(u\right)}\right\}}_{u=1}^{u}$ to reduce network redundancy; ${\left\{{\tilde{S}}^{\left(v\right)}\right\}}_{v=1}^{v}$ denotes the denoised similarity matrices by further denoised diffusion; $X\in R^{N\times D}$ denotes common attribute feature representation learned by joint decomposition with graph regularization, $Y\in R^{N\times D}$ denoted topological feature representation learned by feature extraction based on KG, and $Z\in R^{N\times D}$ denotes the feature representation fused by iAFF.

**FIGURE 1:**
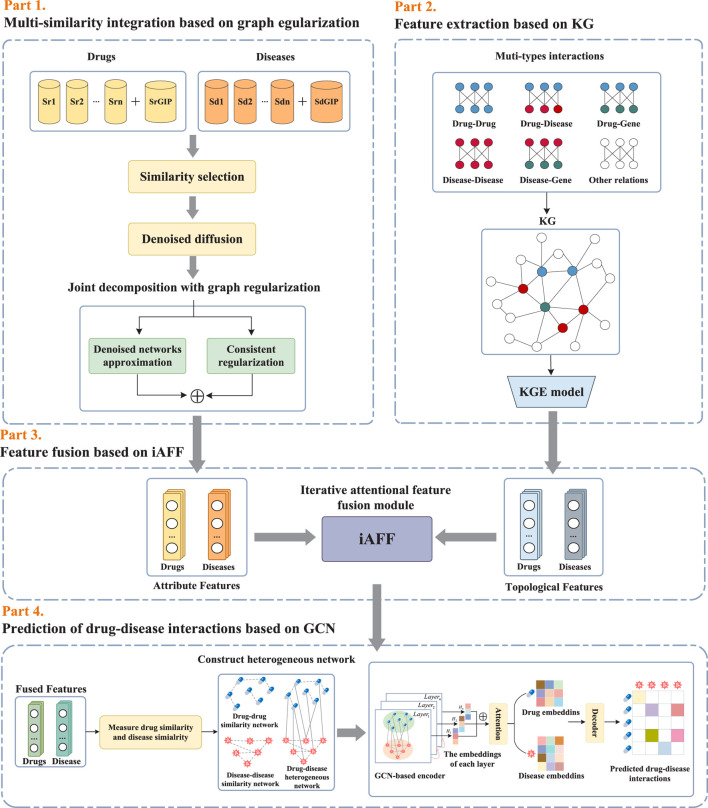
The workflow of the proposed KGRDR.

As illustrated in Figure 1, the proposed framework KGRDR mainly consists of four steps, including multi-similarity integration based on graph regularization, feature extraction based on KG, feature fusion based on iAFF, and drug-disease interaction prediction based on GCN. In the first step, a graph regularized integration method is used to fuse and compute common attribute feature representation $X\in R^{N\times D}$ for multiple denoised similarity networks ${\left\{{\tilde{S}}^{\left(v\right)}\right\}}_{v=1}^{v}$ . In the second step, knowledge graph embedding is used to extract global topological feature representation $Y\in R^{N\times D}$ of drug and disease entities. In the third step, an iterative attention feature fusion method iAFF is used to effectively integrate the attribute feature representation X and topological feature representation Y . In the fourth step, the fused drug and disease feature vectors $Z\in R^{N\times D}$ are fed into the GCN model to predict new drug-disease interactions.

### 2.2 Multi-similarity integration based on graph regularization

Considering that noise and redundant information in the multi-similarity networks significantly affect the model’s prediction performance, a graph regularized integration approach (Zhang et al., 2022b) is used to denoise and fuse the multi-similarity information of drugs and diseases. This method primarily consists of denoised diffusion and joint decomposition. The denoised diffusion module is used to denoise multiple similarity networks, while the joint decomposition module fuses the denoised networks. Additionally, to eliminate similarity networks with minimal information and excessive redundancy, a similarity selection operation is employed to pre-screen the similarity matrices before the graph regularized integration step.

#### 2.2.1 Drug and disease similarity data

The pairwise similarity data of drug-related and disease-related entities used in this study are obtained from SND (Jarada et al., 2021) and SCMFDD_S (Zhang et al., 2018) datasets. SND includes ten drug-related similarity data, fourteen disease-related similarity data, and drug-disease interaction data. SCMFDD_S contains five drug-related similarity data, one disease-related similarity data, and drug-disease interaction data. The pairwise similarity values between drugs and diseases range from 0 to 1.

In addition to the above similarity data, we also calculate Gaussian interaction profile kernel similarity of drugs and diseases based on known drug-disease interaction information Van Laarhoven et al. (2011). Drug r is represented by a binary feature vector f(r) , where the absence or presence of a disease interaction is encoded by 0 or 1, respectively. Similarly, Disease d is represented by a binary feature vector g(d) , where the absence or presence of a drug interaction is encoded by 0 or 1, respectively. The profile similarity values for drug pairs and disease pairs were calculated as shown in Equations 1, 2.

$$S_{r\left(GIP\right)}\left(r_{1},r_{2}\right)=exp\left(\frac{-\gamma n_{r}\|f\left(r_{1}\right)-f\left(r_{2}\right){\|}^{2}}{\overset{n_{r}}{\underset{i=1}{\sum }}|f\left(r_{i}\right){|}^{2}}\right)$$ (1)

$$S_{d\left(GIP\right)}\left(d_{1},d_{2}\right)=exp\left(\frac{-\gamma n_{d}\|g\left(d_{1}\right)-g\left(d_{2}\right){\|}^{2}}{\overset{n_{d}}{\underset{j=1}{\sum }}|g\left(d_{j}\right){|}^{2}}\right)$$ (2)

where the parameter γ controls the kernel bandwidth, $n_{r}$ and $n_{d}$ are the total number of drugs and diseases, $\left|f\left(r_{i}\right)\right|$ is the number of diseases that interact with drug $r_{i}$ , $\left|g\left(d_{j}\right)\right|$ is the number of drugs that interact with disease $d_{j}$ . Here, γ was simply set to 1 as indicated by Van Laarhoven et al. (2011).

#### 2.2.2 Similarity selection

The quality, richness and correlation of drug-related and disease-related similarity matrices vary considerably (Jarada et al., 2021). Data inconsistency and redundancy can introduce noise. This study adopted an effective method, introduced by Olayan et al. (2018), to select the most informative and less redundant drug and disease similarity subset. The heuristic similarity selection process consists of four parts: calculating the average entropy of similarity matrices, ranking the matrices according to their average entropy values, calculating the similarity measure between similarity matrices from different data sources, and eliminating redundant similarity matrices.

In this study, we use the calculated profile similarity and similarities from SND and SCMFDD_S datasets to perform the similarity selection. The similarity selection step extracts a subset ${\left\{S^{\left(v\right)}\right\}}_{v=1}^{\text{v}}$ of the similarity matrices that is both highly informative and minimally redundant, V denotes the number of similarity matrix.

#### 2.2.3 Diffusion to denoise matrices

Some noise may still exist in the extracted similarity matrices. To address this problem, we apply denoised diffusion (Wang et al., 2018) to further reduce the noise in the similarity matrices ${\left\{S^{\left(v\right)}\right\}}_{v=1}^{\text{v}}$ obtained by similarity selection. The denoised similarity matrix ${\left\{{\tilde{S}}^{\left(v\right)}\right\}}_{v=1}^{v}$ is computed as Equation 3.

$${\tilde{S}}^{\left(v\right)}=\left(1-\alpha \right)U^{\left(v\right)}\left(\Sigma {\left(I-\alpha \Sigma^{2}\right)}^{-1}\right){\left(U^{\left(v\right)}\right)}^{-1}$$ (3)

where ${\tilde{S}}^{\left(v\right)}\in R^{N\times N}$ represents the $v^{th}$ denoised similarity matrix of size N×N , α∈(0,1) is a hyperparameter that increases the strength of self-similarity, $U^{\left(v\right)}$ is the matrix with eigenvectors of $S^{\left(v\right)}$ as its columns, I is the N×N identity matrix and ∑ is a diagonal matrix with eigenvalues of $S^{\left(v\right)}$ as its entries. Through the above iterative process, denoised matrix ${\tilde{S}}^{\left(v\right)}$ can be computed for each similarity matrix, which is used in the next joint decomposition step.

#### 2.2.4 Joint decomposition with graph regularization

In order to fuse the denoised similarity matrices in ${\left\{{\tilde{S}}^{\left(v\right)}\right\}}_{v=1}^{v}$ , we employ a graph regularized integration method (Zhang et al., 2022b), which combines joint decomposition (Cho et al., 2016) with graph regularization to learn a common attribute feature representation $X\in R^{N\times D}$ from denoised similarity matrix subsets ${\left\{{\tilde{S}}^{\left(v\right)}\right\}}_{v=1}^{v}$ .

The joint decomposition method can analyze multiple networks uniformly, capture the inconsistencies between networks, and explore the correlations between networks. Specifically, for the denoised similarity subset ${\left\{{\tilde{S}}^{\left(v\right)}\right\}}_{v=1}^{v}$ , the common feature matrix X is used to represent the common components between networks, and the specific feature matrix $W^{\left(v\right)}$ is used to represent the specific components for network v. Each denoised matrix ${\tilde{S}}^{\left(v\right)}$ is approximated by a reconstruction matrix ${\hat{S}}^{\left(v\right)}$ with ${\hat{S}}^{\left(v\right)}=softmax\left(X^{\text{T}}W^{\left(v\right)}\right)$ . The approximation error of ${\tilde{S}}^{\left(v\right)}$ and ${\hat{S}}^{\left(v\right)}$ is measured by the Kullback–Leibler (KL) divergence and described as Equation 4.

$$\begin{array}{cc} L_{\mathrm{appr}} & =\overset{V}{\underset{v=1}{\sum }}KL\left({\tilde{S}}^{\left(v\right)}\|{\hat{S}}^{\left(v\right)}\right)\\ & =\frac{1}{N}\overset{V}{\underset{v=1}{\sum }}\overset{N}{\underset{i,j=1}{\sum }}{\tilde{s}}_{ij}^{\left(v\right)}\log{\tilde{s}}_{ij}^{\left(v\right)}\\ & -\frac{1}{N}\overset{V}{\underset{\nu =1}{\sum }}\overset{N}{\underset{i,j=1}{\sum }}{\tilde{s}}_{ij}^{\left(\nu \right)}\left[x_{i}^{\text{T}}w_{j}^{\left(\nu \right)}-\log\left(\overset{N}{\underset{j^{\prime }=1}{\sum }}\exp\left\{x_{i}^{\text{T}}w_{j^{\prime }}^{\left(\nu \right)}\right\}\right)\right] \end{array}$$ (4)

in which $x_{i}$ are column vectors of X , representing a common feature vector of node i in the $v^{th}$ network, and $w_{j}^{\left(v\right)}$ are column vectors of $W^{\left(v\right)}$ , representing a specific feature vector of node j in the $v^{th}$ network.

Moreover, to ensure that the common feature X can accurately represent the refined structural information from ${\left\{{\tilde{S}}^{\left(v\right)}\right\}}_{v=1}^{\text{v}}$ , a graph Laplacian regularization term $L_{\mathrm{reg}}$ is introduced on X . Specifically, the consistency of all node pairs is defined as Equation 5.

$$\begin{array}{cc} L_{\mathrm{reg}} & =\overset{V}{\underset{v=1}{\sum }}\overset{N}{\underset{i,j=1}{\sum }}\|x_{i}-x_{j}{\|}^{2}{\tilde{s}}_{ij}^{\left(v\right)}\\ & =\overset{V}{\underset{\nu =1}{\sum }}\left[Tr\left(X{\tilde{D}}^{\left(\nu \right)}X^{\text{T}}\right)-Tr\left(X{\tilde{S}}^{\left(\nu \right)}X^{\text{T}}\right)\right]\\ & =\overset{V}{\underset{v=1}{\sum }}Tr\left(X{\tilde{L}}^{\left(v\right)}X^{\text{T}}\right) \end{array}$$ (5)

in which ${\tilde{D}}^{\left(v\right)}$ denotes the diagonal degree matrix of ${\tilde{S}}^{\left(v\right)}$ with ${\tilde{L}}^{\left(v\right)}={\tilde{D}}^{\left(v\right)}-{\tilde{S}}^{\left(v\right)}$ is the graph Laplacian matrix and Tr(⋅) denotes the trace of a matrix.

The objective function is described as Equation 6.

$$\begin{array}{cc} \underset{{\left\{{\tilde{W}}^{\left(v\right)}\right\}}_{v=1}^{V},X}{\min}L & =L_{\mathrm{appr}}+L_{\mathrm{reg}}\\ & =\overset{V}{\underset{v=1}{\sum }}\left[KL\left({\tilde{S}}^{\left(v\right)}\|{\hat{S}}^{\left(v\right)}\right)+\lambda Tr\left(X{\tilde{L}}^{\left(v\right)}X^{\text{T}}\right)\right] \end{array}$$ (6)

where λ≥0 is the regularization parameter, X is regarded as the common attribute feature representation shared across all similarity matrices. The dimension of the feature representation X directly affects the integration capacity of multi-similarity networks and the performance of downstream prediction tasks. To investigate its effect on model predictions, we subsequently conducted a parameter sensitivity analysis on the feature representation dimension.

### 2.3 Feature extraction based on KG

In this study, we used the Drug Repurposing Knowledge Graph (DRKG) (Ioannidis et al., 2020) to learn topological features of drugs and diseases. DRKG is specifically designed for drug repurposing and includes entities such as drugs, diseases, and genes, along with their relationships. Knowledge graph embedding maps these entities and relationships into a low-dimensional vector space, preserving both structural and semantic information. In this study, we applied the ComplEx knowledge graph (KG) embedding method (Trouillon et al., 2016) to learn embedding representations.

#### 2.3.1 Knowledge graph construction

DRKG included 97,238 entities belonging to 13 entity types and 5,874,261 triples belonging to 107 edge types. The types of entities and relationships included in the knowledge graph are as follows.

• Entities: drugs, diseases, genes, compounds, etc.
• Relations: drug-target, gene-disease, drug-disease, etc.

DRKG is composed of entity-relation-entity triples. For example, the triple (DB00512, Compound:Disease, C0157749) indicates that drug DB00512 interacts with disease C0157749. In the knowledge graph, entities are represented as nodes, and relations are represented as edges from the subject entity node to the object entity node. We removed all the triples of drug-disease relationship in DRKG that were not present in the benchmark dataset (Jarada et al., 2021; Zhang et al., 2018), added new triples of drug-disease relationship that existed in dataset to DRKG, and regarded the obtained triples in DRKG as the KG dataset.

#### 2.3.2 Knowledge graph embedding

As shown in Figure 1, we employed the widely adopted KGE method, ComplEx (Trouillon et al., 2016), to derive KG-based feature representations for each entity and relationship in the DRKG. ComplEx models entities and relations by embedding them into complex domains, which can capture the asymmetric relationship between triples (drug-disease-relationship) while preserving the vector representation, thereby more effectively handling the drug-disease interaction prediction problem. The process is as follows.

• Embedding Initialization: The dataset contains $n_{r}$ drugs and $n_{d}$ diseases. For each drug $r_{i}$ , we learn the complex vector $e_{s}\in C^{k}$ of $r_{i}$ , and $C^{k}$ represents a complex vector space with k dimensions. Similarly, for each disease $d_{j}$ , learn the complex vector $e_{o}\in C^{k}$ of $d_{j}$ . For the drug-disease interaction relationship r , we learn the complex vector $w_{r}\in C^{k}$ .
• Predicting drug-disease interaction scores: A scoring function is used to measure the interaction strength between the learned complex embedding vectors of drugs and diseases, thereby predicting potential drug-disease interactions. The scoring function is a core component of the ComplEx embedding method. By training and optimizing the scoring function, the embedding vectors of drugs and diseases are adjusted and refined. The scoring function for the triple is described as Equation 7.

ϕr,s,o;Θ=Re<wr,es,e¯o>=Re∑k=1Kwrkeske¯ok=〈Rewr,Rees,Reeo〉+〈Rewr,Imes,Imeo〉+〈Imwr,Rees,Imeo〉−Imwr,Imes,Reeo (7)

where Θ denotes the model parameters, $w_{r}$ is the embedding vector of the relation, $e_{s}$ is the subject vector of the relation, ${\bar{e}}_{o}$ is the conjugate object vector of the relation, Re(x) means to take the real vector component of x , Im(x) means to take the imaginary vector component of x . In the complex space, $<e_{o},e_{s}>=\bar{<e_{s},e_{o}>}$ , so $Re\left(<e_{o},e_{s}>\right)$ is symmetric, while $Im\left(<e_{o},e_{s}>\right)$ is antisymmetric. The score for the triple (s,r,o) is calculated as the product of the conjugate vector of the relation r and the vectors representing the subject s and object o , with the real part of the final result retained. The predicted probability of interaction that the triple (s,r,o) existed in knowledge graph is calculated by the logistic inverse link function defined in Equation 8.

$$P\left(Y_{\mathrm{rso}}=1\right)=\sigma \left(\phi \left(r,s,o;\Theta \right)\right)$$ (8)

• Model training and optimization: In order to learn appropriate embedding representations of drugs and diseases, the ComplEx model optimizes the embedding representation through negative sampling and loss function. The goal of the loss function is to maximize the prediction score of the true drug-disease interaction pair while minimizing the score of the negative sample pair. The loss function of the model is defined as Equation 9.

$$\underset{\Theta }{\min}\underset{r\left(s,\rho \right)\in \Omega }{\sum }\log\left(1+\exp\left(-Y_{r,\rho }\phi \left(s,r,\sigma ,\Theta \right)\right)\right)+\lambda \|\Theta {\|}_{2}^{2}$$ (9)

where λ is a hyper-parameter introduced in the study (Trouillon et al., 2016).

• Extracting features: Using the trained model, the embedding vectors of drug and disease entities are extracted as their global topological feature representation $Y\in R^{N\times D}$ .

### 2.4 Feature fusion based on iAFF

**FIGURE 2:**
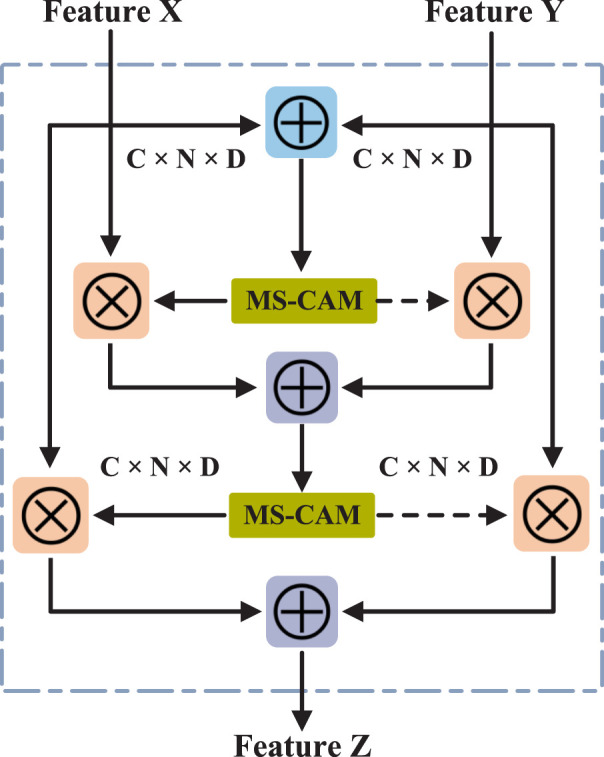
The structure of iterative attention feature fusion.

In this study, we used the iterative attention feature fusion (iAFF) method (Dai et al., 2021) to fuse the attribute feature $X\in R^{N\times D}$ obtained from the graph regularized integration module with the topological feature $Y\in R^{N\times D}$ learned from the knowledge graph embedding. The iAFF updates the feature representation iteratively and uses the Multiscale Channel Attention Module (MS-CAM) to learn feature information with different scale in each channel and calculate the attention weight of each channel feature. These attention weights are multiplied element-wise with the original features to achieve channel-level feature enhancement. As shown in Figure 2, the input feature representations X and Y are extracted by CNN to obtain intermediate features with C channels and feature maps of size N×D . First, we perform the initial feature fusion X⊕Y on the input features X and Y . Next, to achieve a more comprehensive perception of the input feature map, the MS-CAM module is used to calculate attention weights of X⊕Y , assigning different weights to features X and Y . The detailed calculation of the attention feature fusion is defined as Equation 10.

$$X⊎Y=M\left(X⊕Y\right)⊗X⊕\left(1-M\left(X⊕Y\right)\right)⊗Y$$ (10)

where ⊎ denotes the disjoint union operation, ⊕ denotes broadcasting addition, ⊗ denotes the element-wise product operation, M(⋅) denotes the generation of attention weights using the MS-CAM module and the dashed line indicates the operation 1−M(X⊕Y) .

Finally, the attention-fused feature X⊎Y is iteratively used in the MS-CAM module to calculate new attention weights, assigning defferent weights to feature X and Y . This two-stage calculation method is called iterative attention feature fusion (iAFF). The iteratively attention-fused feature Z is defined as Equation 11.

$$Z=M\left(X⊎Y\right)⊗X⊕\left(1-M\left(X⊎Y\right)\right)⊗Y$$ (11)

### 2.5 Prediction of drug-disease interactions based on GCN

Recently, graph convolutional networks (GCNs) (Kipf and Welling, 2016) have attracted increasing attention and have been widely applied to various drug repositioning prediction tasks in an end-to-end manner (Peng et al., 2022). In this study, the drug and disease feature vectors obtained through feature fusion, along with known drug-disease interactions, are fed into a graph convolutional neural network (Cai et al., 2021) to predict candidate drug-disease interactions.

#### 2.5.1 Construction of the drug–disease heterogeneous network

Using the fused drug and disease feature matrix, the similarity matrix R and D are calculated based on the Euclidean distance. Subsequently, the drug-disease heterogeneous network is constructed using the drug similarity matrix R, the disease similarity matrix D, and the drug-disease interaction matrix.

The drug–drug similarity matrix is denoted as a graph $G_{r}$ with N drugs, and its adjacency matrix $A^{r}\in R^{N\times N}$ is composed of the drug similarity matrix $S^{r}$ . Specifically, if drug is the $r_{j}$ nearest neighbor of drug $r_{i}$ based on drug similarity matrix $S^{r}$ , then the (i,j) th entry of $A^{r}$ is $S_{ij}^{r}$ ; otherwise $A_{ij}^{r}=0$ . topk denotes the number of K nearest neighbors of each drug or each disease. Similarly, the disease-disease similarity matrix is denoted as a graph $G_{d}$ with M diseases, and its adjacency matrix $A^{d}\in R^{M\times M}$ is composed of disease similarity matrix $S^{d}$ . Specifically, if disease $d_{j}$ is the topk nearest neighbor of disease $d_{i}$ based on disease similarity matrix $S^{d}$ , then the (i,j) th entry of $A^{d}$ is $S_{ij}^{d}$ ; otherwise $A_{ij}^{d}=0$ . The drug–disease interaction matrix is denoted as a graph G with N drugs and M diseases, and its adjacent matrix is $A\in {\left\{0,1\right\}}^{N\times M}$ . $A_{ij}=1$ if a drug $r_{i}$ is associated with a disease $d_{j}$ . $A_{ij}=0$ if the association between drug $r_{i}$ and disease $d_{j}$ is unknown or unobserved.

#### 2.5.2 Feature extraction based on GCN

GCN is a multilayer connected neural network architecture and is used to learn low-dimensional representations of nodes from graph-structured data. In this study, we employed a heterogeneous information fusion graph convolutional network model (Cai et al., 2021) to predict drug-disease associations. The strategy of fusing intra-domain features and extra-domain features is used by this method to improve the prediction performance. Specifically, the intra-domain embeddings of drugs and diseases are first extracted using the drug-drug similarity network and the disease-disease similarity network. Then, the inter-domain embeddings of drugs and diseases are extracted using the drug-disease association network. Finally, the inter-domain embeddings and intra-domain embeddings are fused to obtain the final embedding representations of drugs and diseases.

First, we initialize the embeddings of drugs and diseases as Equation 12.

$$\begin{array}{c} H^{0}=\left[\begin{array}{c} H_{r}^{0}\\ H_{d}^{0} \end{array}\right]=\left[\begin{array}{cc} S^{r} & 0\\ 0 & S^{d} \end{array}\right]\in R^{\left(N+M\right)\times \left(N+M\right)} \end{array}$$ (12)

Second, the intra-domain feature extraction module is defined as Equation 13.

$$\begin{array}{c} {\hat{H}}^{l+1}=\left[\begin{array}{c} {\hat{H}}_{r}^{l+1}\\ {\hat{H}}_{d}^{l+1} \end{array}\right]=\left[\begin{array}{c} GCN\left(A^{r},H_{r}^{l},W_{r}^{l}\right)\\ GCN\left(A^{d},H_{d}^{l},W_{d}^{l}\right) \end{array}\right] \end{array}$$ (13)

where ${\hat{H}}_{r}^{l+1}\in R^{N\times k}$ is the drug intra-domain output features at the l th-layer, ${\hat{H}}_{d}^{l+1}\in R^{M\times k}$ is the disease intra-domain output features at the l th-layer, is the drug input embeddings at the l th-layer, $H_{r}^{l}$ is the disease input embeddings at the l th-layer and $W_{r}^{l}\in R^{k\times k}$ and $W_{d}^{l}\in R^{k\times k}$ are trainable matrices of the l th-layer intradomain feature extraction module. Graph convolution operation is denoted as GCN(A,H,W) , and is formulated as Equation 14.

$$GCN\left(A,H,W\right)=\sigma \left(D^{-\frac{1}{2}}{AD}^{-\frac{1}{2}}HW\right)$$ (14)

where $D=diag\left(\sum_{j}A_{ij}\right)$ and σ(⋅) is a ReLU (Nair and Hinton, 2010) activation function.

The inter-domain feature extraction module for message passing between drugs and diseases is composed of a bilinear aggregator (BA) and a traditional GCN aggregator (AGG). Specifically, for a drug $r_{i}$ , its drug inter-domain feature extraction module is defined as Equation 15.

$${\overset{∼}{H}}_{r_{i}}^{l+1}=\sigma \left(\alpha^{l}\frac{\underset{j}{\sum }\left(H_{d_{j}}^{l}W^{l}⊙H_{r_{i}}^{l}W^{l}\right)A_{ij}}{\underset{j}{\sum }A_{ij}}+\left(1-\alpha^{l}\right)\frac{\underset{j}{\sum }H_{d_{j}}^{l}W^{l}A_{ij}}{\underset{j}{\sum }A_{ij}}\right)$$ (15)

where ⊙ is an element-wise product, ${\overset{∼}{H_{r_{i}}}}^{l+1}\in R^{k}$ is the l th-layer drug inter-domain output feature of drug $r_{i}$ , $W^{l}\in R^{k\times k}$ is a trainable matrix and $\alpha^{l}\in R$ is a trainable scalar used to balance the importance between BA and the traditional GCN aggregator. For disease $d_{j}$ , its disease inter-domain feature extraction module is defined in the same way as the drug inter-domain feature extraction module, using ${\overset{∼}{H}}_{d_{j}}^{l+1}\in R^{k}$ to represent the l th-layer of disease inter-domain output features of disease $d_{j}$ .

Finally, the intra-domain features and inter-domain features are merged as Equation 16.

$$\begin{array}{c} \left.H^{l+1}={\hat{H}}^{l+1}+{\tilde{H}}^{l+1}+H^{l}=\left[\begin{array}{c} {\hat{H}}_{r}^{l+1}+{\tilde{H}}_{r}^{l+1}+H_{r}^{l}\\ {\hat{H}}_{d}^{l+1}+{\tilde{H}}_{d}^{l+1}+H_{d}^{l} \end{array}\right.\right] \end{array}$$ (16)

where $H^{l+1}$ is the l th-layer output embeddings of the nodes (drugs and diseases). The embeddings at different GCN layers capture different levels of information of the input graphs. After the L layer, we obtained L k-dimensional drug and disease embeddings, respectively.

Next, layer attention is introduced into the network architecture to adaptively combine embeddings at different graph convolution layers with an attention mechanism to further improve the prediction performance. Specifically, we paid different attention to convolution layers to integrate embeddings and obtained the final embeddings of drugs and diseases as Equation 17.

$$\begin{array}{c} \left.\left[\begin{array}{c} H_{R}\\ H_{D} \end{array}\right.\right]=\underset{l=1}{\sum }\beta^{\text{l}}H^{\text{l}} \end{array}$$ (17)

where $\beta^{l}\in R$ is auto-learned by neural networks and initialized as 1/L, which denotes the contributions of the embeddings at different convolution layers to the feature embeddings. $H_{R}\in R^{N\times k}$ is the feature embeddings of drugs, and $H_{D}\in R^{M\times k}$ is the final embeddings of diseases.

#### 2.5.3 Drug-disease interaction prediction

To reconstruct the associations between drugs and diseases, the decoder $f\left(H_{R},H_{D}\right)$ is formulated as Equation 18.

$$\hat{A}=f\left(H_{\text{R}},H_{\text{D}}\right)=sigmoid\left(H_{\text{R}}H_{\text{D}}^{\text{T}}\right)$$ (18)

where $\hat{A}\in R^{N\times M}$ is the predicted probability score matrix. The predicted score for the association between drug $r_{i}$ and disease $d_{j}$ is given by the corresponding (i,j) th entry of $\hat{A}$ .

The parameters are learned by minimizing the weighted binary cross entropy loss as Equation 19.

$$loss=-\frac{1}{N\times M}\left(\gamma \times \underset{\left(i,j\right)\in S^{+}}{\sum }\log{\hat{A}}_{ij}+\underset{\left(i,j\right)\in S^{-}}{\sum }\left(1-\log{\hat{A}}_{ij}\right)\right)$$ (19)

where N denotes the number of drug nodes, M denotes the number of disease nodes, (i,j) denotes the pair of drug $r_{i}$ and disease $d_{j}$ , $S^{+}$ denotes the set of all known drug–disease association pairs and $S^{-}$ represents the set of all unknown or unobserved drug–disease association pairs. The balance factor $\gamma =\frac{\left|S^{-}\right|}{\left|S^{+}\right|}$ is used to reduce the impact of data imbalance, where $\left|S^{-}\right|$ and $\left|S^{+}\right|$ are the number of pairs in $S^{+}$ and $S^{-}$ .

Finally, the model is optimized using the Adam optimizer (Diederik, 2014) and the weights are initialized as described in Glorot and Bengio (2010).

## 3 Results and discussion

### 3.1 Datasets

Two benchmark datasets containing drug-related information similarity, disease-related information similarity, and drug-disease interaction are used in this study. The detailed information of the two datasets is shown in Table 1.

**TABLE 1:** Detailed information of benchmark datasets.

Datasets	No. drugs	No. disease	No. interactions	Sparsity	No. drug similarities	No. disease similarities
SND (Jarada et al., 2021)	867	803	8,684	0.9875	10	14
SCMFDD_S (Zhang et al., 2018)	269	598	18,416	0.8855	5	1

#### 3.1.1 SND dataset

The SND benchmark dataset was assembled from various biological and biomedical data sources. Drug-disease interaction data was collected from two widely used data sources, namely, DrugBank (Wishart et al., 2018) and repoDB (Brown and Patel, 2017). The dataset contains 867 FDA-approved drugs, 803 diseases, and 8,684 clinically reported and/or experimentally validated drug-disease interactions with 98.75% sparsity.

SND contains ten drug similarity data: (1) target interaction similarity, (2) side effect similarity, (3) chemical structure similarity, (4) GO molecular function similarity, (5) GO biological process similarity, (6) GO cellular component similarity, (7) metabolism enzyme similarity, (8) protein sequence similarity, (9) ATC code similarity and (10) drug interaction similarity.

Moreover, the dataset contains fourteen disease similarity (1) curated gene similarity, (2) HPO gene similarity, (3) literature gene similarity, (4) curated variant similarity, (5) literature variant similarity, (6) microRNA similarity, (7) lncRNA similarity, (8) HPO phenotype similarity, (9) IS-A taxonomy similarity (10) information-theoretic similarity, (11) GO term similarity (12) implicit semantic similarity, (13) semantic & functional similarity, and (14) association ontology similarity.

#### 3.1.2 SCMFDD_S dataset

The SCMFDD_S benchmark dataset are collected from the literature (Zhang et al., 2018). The drug-disease interaction data include 18,416 known drug-disease interactions between 269 drugs and 598 diseases from CTD (Davis et al., 2017), with a sparsity of 88.55%.

SCMFDD_S contains five drug similarity data: (1) target interaction similarity, (2) metabolism enzyme similarity, (3) drug interaction similarity, (4) pathway similarity, and (5) chemical substructure similarity. Information on drug targets, enzymes, and related aspects is sourced from DrugBank (Wang et al., 2010).

Disease similarity data based on the MeSH descriptor is contained in SCMFDD_S. The MeSH descriptors of the disease can be represented as a hierarchical directed acyclic graph (DAG), and the DAG structure can be used to calculate the similarity between two diseases (Meng et al., 2022).

### 3.2 Method comparisons

To validate the effectiveness of our approach in predicting drug-disease associations, we compared KGRDR with six state-of-the-art drug repositioning methods based on recommender systems and GCNs, including LAGCN (Yu et al., 2021), DRHGCN (Cai et al., 2021), DRWBNCF (Meng et al., 2022), GCNAT (Sun et al., 2022), SMGCL (Gao et al., 2023), and DRAGNN (Meng et al., 2024). These methods are described in detail as follows.

• LAGCN is a layered attention graph convolutional network, which is used for the drug–disease associations prediction.
• DRHGCN uses GCN to extract inter-domain and intra-domain feature information of drugs and diseases, thereby finding new drug indications based on different network topology information of drugs and diseases in different domains.
• DRWBNCF is a neural collaborative filtering method that proposes a new weighted bilinear graph convolution operation to integrate the information of the known drug–disease association, drug’s and disease’s neighborhood, and neighborhood interaction into a unified representation to infer novel potential drug–disease associations.
• GCNAT is a deep learning algorithm that combines graph convolutional networks (GCN) and graph attention networks (GAT). After building a heterogeneous network, this approach combines the embeddings of multiple convolutional layers using a graph attention layer on a constructed heterogeneous network and assigns different weights to predict new metabolite-disease associations.
• SMGCL is a graph contrastive learning method based on similarity measurement. It introduces graph contrastive learning methods and jointly trains node representations to maximize consistency, thereby overcoming the problem of sparse supervision signals in traditional graph neural network methods and enhancing the predictive ability of drug-disease associations.
• DRAGNN is a local information weighted enhancement method that improves the effectiveness of target node information collection by combining the attention mechanism and omitting self-node information aggregation, thereby improving the prediction performance of the model.

These competing methods with the optimal parameters suggested in the original papers are compared with KGRDR using 10-fold cross-validation. Furthermore, we conducted parameter analysis and selected the best parameters for KGRDR.

### 3.3 Parameter settings

There are multiple parameters in KGRDR which can impact the model performance. According to the literature (Zhang et al., 2022b), the number of neighbors K in the graph regularization integration module is set to 20, the surfing parameter α is set to 1, the regularized parameter γ is set to 10, and the embedding feature dimension is set to 500. We initialize the embedding dimension of KG to 500, set the learning rate to 0.1, and set the regularization coefficient to 1.00E-07. GCN adopts a three-layer architecture with 64 hidden units in each layer. In terms of variable settings, referring to the settings of Cai et al. (2021), the regular loss rate of 0.4, the edge loss rate of 0.2, the learning rate of 0.05, the topk of 15, and the maximum number of training epochs for all experiments is 4,096. The hyperparameters of LAGCN, DRHGCN, DRWBNCF, GCNAT, SMGCL and DRAGNN are selected according to the optimal values provided by their publications. We use 10-fold cross validation to evaluate model parameters and grid search to select the best hyperparameter settings. Since the embedding dimension directly impacts the learned representation of the fused feature vectors and knowledge graph embedding vectors, while the channel scaling ratio reflects the compression of channel information, we tested the above parameters: graph regularized integration and KG embedding feature dimensions within the range of ∈100,200,300,400,500,600 , channel scaling ratios within the range of ∈2,4,8,16,32,64 in iterative attention feature fusion. Table 2 provides the details of the parameter settings.

**TABLE 2:** Hyperparameter settings.

Parameter	Setting
epoch	4,096
embedding_dim	128
learning rate	5 × 10^–2^
layer_num	2
topk	15
dropout	0.4
edge_dropout	0.2
optimizer	Adam
GR_dim	500
KG_dim	500
KG_model_name	ComplEx
KG_lr	0.1
KG_regularization	1 × 10^–7^

### 3.4 Performance evaluation

In this study, we conducted 10-fold cross-validation on two benchmark datasets to evaluate the performance of KGRDR. During the 10-fold cross-validation, we randomly selected 10% of the known drug-disease associations and 10% of the unknown associations in the dataset as the testing set; the remaining 90% of clinically reported drug-disease association and unknown drug-disease associations pairs were used to train the prediction model. The area under the receiver operating characteristic curve (AUROC) and the area under the precision–recall curve (AUPR) has been widely used in bioinformatics research (Zhang et al., 2020a; Zhang et al., 2020b). We adopt these two metrics to evaluate the overall performance of KGRDR and compare it with six state-of-the-art association prediction methods.

As shown in Table 3, on the SND dataset, KGRDR finally achieved an AUROC of 0.9864, which is 2.09% higher than the second-ranked DRHGCN; KGRDR achieved an AUPR of 0.7915, which is 11.69% higher than the second-ranked DRHGCN. It is worth noting that KGRDR also achieved the highest AUROC and AUPR on the SCMFDD_S dataset. The benchmark comparison results of the two datasets show that KGRDR outperforms six state-of-the-art prediction models. In particular, the results of each 10-fold cross-validation are basically consistent, which shows that our model shows convincing performance and high robustness.

**TABLE 3:** Comparison of KGRDR with the state-of-the-art methods.

Method	SND dataset		SCMFDD_S dataset	
	AUROC	AUPR	AUROC	AUPR
LAGCN	0.8890	0.3791	0.8695	0.2502
DRHGCN	0.9655	0.6915	0.8667	0.5345
DRWBNCF	0.9278	0.6630	0.8480	0.4761
GCNAT	0.7578	0.2539	0.8240	0.3070
SMGCL	0.9343	0.4434	0.8665	0.5057
DRAGNN	0.8560	0.3016	0.8059	0.4190
KGRDR	**0.9864**	**0.8085**	**0.8739**	**0.5470**

The best results are highlighted in bold.

The excellent prediction performance of KGRDR can be attributed to learn the feature representations of drugs and diseases from different perspectives and fuse these features to improve the prediction performance. First, KGRDR considers14 the information of drugs and diseases from multiple similarity perspectives, and uses a graph regularized integration method to fuse the different similarity information of drugs and diseases, which can obtain more comprehensive common attribute feature information. Then, KGRDR uses the existing drug-disease interaction data and combines all related entities in KG to enrich the global topological representation of drugs and diseases. Finally, the attention feature fusion method is used to fuse these two features of information, which can more effectively represent the entity characteristics of drugs and diseases, thereby further improving the performance of the drug-disease interaction prediction model.

### 3.5 Parameter sensitivity analysis

To further analyze KGRDR, we studied the impact of some parameters on model performance. The dimension of embedding directly affects the representation ability of the fused feature vector and the knowledge graph embedding vector. Increasing the embedding dimension can effectively encode more feature information. However, exceeding a certain range will lead to overfitting, which will reduce the model performance. The channel scaling ratio reflects the compression of channel information. Reducing the channel scaling ratio makes the number of channels of the feature map larger, which means that the model can retain more feature information. However, having an excessive number of channels can lead to overfitting, which will affect the model performance. Therefore, we conducted parameter sensitivity analysis on the two parameters to select the optimal parameters for KGRDR. All the following studies were conducted using 10-fold cross validation experiments on the SND benchmark dataset in Table 1.

#### 3.5.1 Effect of the dimension of embedding

**FIGURE 3:**
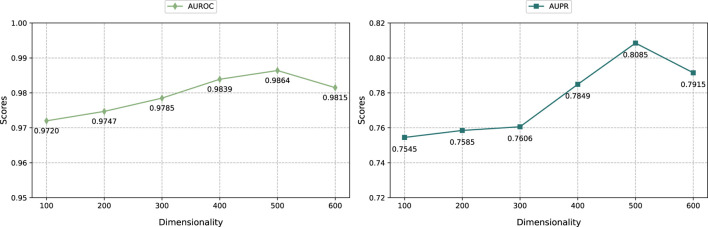
The impact of different embedding dimensions.

As shown in Figure 3, we selected the embedding dimensions of {100, 200, 300, 400, 500, 600} to adjust the graph regularized integration module and the KG embedding representation module, and evaluated them through the AUROC and AUPR indicators. The result shows that when the embedding dimension is 500, the performance of the model reaches optimal. Specifically, as the embedding dimension increases from 100 to 500, the model prediction performance improves significantly. However, when further increasing the embedding dimension to 600, the model performance decreases. This shows that within a certain range, increasing the embedding dimension can effectively encode more feature information, thereby improving model performance. However, exceeding the optimal embedding dimension can lead to overfitting and thus degrade prediction performance.

#### 3.5.2 Effect of the parameter channel scaling ratio

**FIGURE 4:**
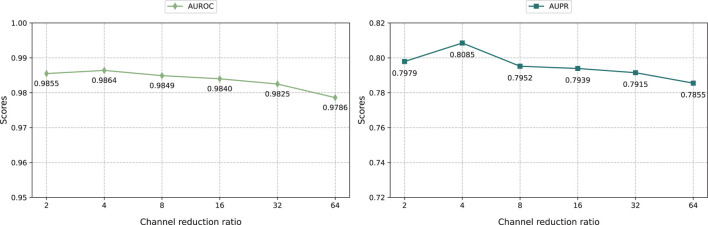
The impact of different channel scaling ratios.

As shown in Figure 4, we evaluated the impact of different channel scaling ratios on the iterative attention feature fusion module effect of drug and disease feature. We changed the channel scaling ratio in the range of {2, 4, 8, 16, 32, 64} and analyzed its impact on the model performance.

The result shows that KGRDR performs best when the channel scaling ratio is set to 4. The smaller the channel scaling ratio, the more channels the intermediate feature map has, which means the model can retain more feature information. Specifically, as the channel scaling ratio increases from 2 to 4, the model prediction performance improves significantly. However, when further increasing the channel scaling ratio to 64, the model performance decreases. This shows that within a certain range, decreasing the channel scaling ratio can effectively encode more feature information, thereby improving model performance. However, exceeding the optimal channel scaling ratio can lead to overfitting, which may degrade prediction performance.

### 3.6 Comparison with the other multi-similarity fusion methods

To further demonstrate the performance of KGRDR in multi-similarity network fusion, we compare it with four methods: SNF-H (Olayan et al., 2018), SNF-NN (Jarada et al., 2021), DeFusion (Wang et al., 2021) and EnMuGR (Zhang et al., 2022b). Among them, SNF-H, DeFusion, and EnMuGR used the same network prediction model (DRHGCN). They were compared with KGRDR on the SND dataset in Table 1. The SNF-NN first compiled and used a large number of drug and disease similarity datasets (SND). The optimal network model (NN) provided by the publication (Jarada et al., 2021) was compared with KGRDR on the SND dataset. The results are as follows.

**FIGURE 5:**
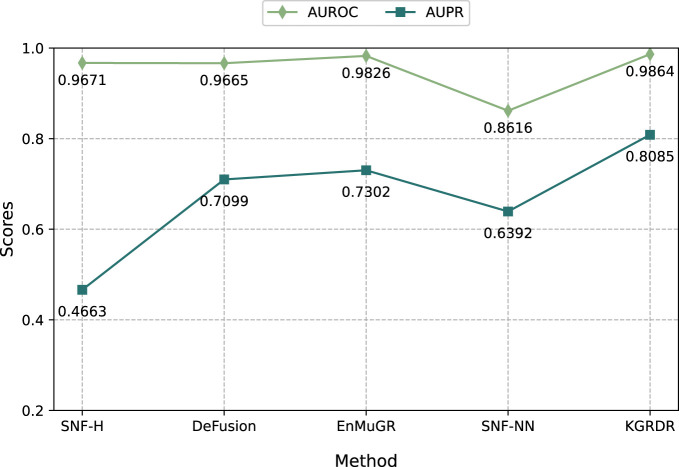
Comparison of multiple similarity network fusion methods.

As shown in Figure 5, when using the same dataset and prediction model, KGRDR achieves the highest AUROC and AUPR values compared to SNF-H, DeFusion, EnMuGR and SNF-NN, indicating that the proposed method better integrates multiple similarity networks, thereby improving the prediction model’ performance.

### 3.7 Ablation study

According to Figure 1, KGRDR mainly consists of four parts: graph regularized integration module, knowledge graph extraction module, iterative attention feature fusion module and graph convolutional network prediction module. In KGRDR, we learned multi-similarity integrated feature representation and knowledge graph-based feature representation. To verify the impact of these two feature representations on model performance, we designed three KGRDR variants and compared them on the SND dataset. The model variants are outlined as follows:

The model variants are summarized as follows.

• KGRDR(w/o KG+GRI) : A variant of KGRDR, which is the original graph convolutional network model based on heterogeneous information fusion, only uses the single similarity information of drugs and diseases for calculation.
• KGRDR(w/o KG) : A variant of KGRDR, which consists only of a feature extraction module based on knowledge graph.
• KGRDR(w/o GRI) : A variant of KGRDR, which consists only of a graph regularized integration module.

Table 4 shows the performance of KGRDR and various variants on the SND dataset. In the 10-fold cross validation, the performance of the combined graph regularized integration module with the knowledge graph extraction module is better than that of using only a single module. This result shows that the weighted attention fusion of the attribute feature information obtained by graph regularized integration and the global topological feature information obtained by knowledge graph extraction helps to improve the prediction performance of KGRDR. Additionally, we validated the predictive ability of KGRDR in drug-disease interactions using the graph regularized integration module and the knowledge graph extraction module separately through local exclusion cross-validation. The experimental result shows that both variants have better predictive performance than the original graph convolutional network model, which indicates that both the graph regularized integration process and the knowledge graph-based feature extraction operation can improve the predictive performance of drug-disease interactions.

**TABLE 4:** Detailed information of benchmark datasets.

Methods	AUROC	AUPR
KGRDR (w/o KG + GRI)	0.9655	0.6915
KGRDR (w/o KG)	0.9826	0.7502
KGRDR (w/o GRI)	0.9845	0.7882
KGRDR	0.9864	0.8085

### 3.8 Case study

To further verify the reliability of KGRDR, the proposed method KGRDR was applied on the SND dataset to learn feature embeddings of drugs and diseases. The learned embeddings were used to predict interaction scores for the unknown drug-disease associations.

In this study, we predicted candidate drugs for two neurodegenerative diseases including Alzheimer’s disease and Parkinson’s disease having a large patient population, high incidence, and no definitive cure. Extensive research has been conducted on the two diseases.

For AD, we focused on the top 10 potential drugs predicted by KGRDR (Table 5) and searched for literature evidence supporting the predictions in the biomedical literature to check the predicted drug-disease associations. The results showed that all 10 candidate drugs (100% success rate) were validated by clinical trials from reliable sources. For example, Escitalopram was originally used to treat major depressive disorder (MDD), generalized anxiety disorder (GAD), and other specific psychiatric disorders such as obsessive-compulsive disorder (OCD). A previous study reported that Escitalopram can improve tau hyperphosphorylation *in vitro*, and inhibition of tau hyperphosphorylation is one of the most promising therapeutic targets for the development of drugs to alleviate Alzheimer’s disease (AD). This was confirmed by an experiment using Escitalopram to alleviate Tau pathological changes in elderly P301L Tau transgenic mice with AD (Wang et al., 2020). The pathogenesis of Alzheimer’s disease (AD) is a complex process, in which the protein toxicity of amyloid β(Aβ) has been identified as a major factor. Phenylbutyric acid has been shown to alleviate the onset of AD by reducing Aβ protein toxicity through its proven chemical chaperone properties or inhibiting histone deacetylase (HDAC) (Baumanns et al., 2023). In addition, the drugs Amitriptyline and Tamoxifen predicted by KGRDR have also been confirmed by biomedical experiments to have significant beneficial effects on aging and impaired AD brains.

**TABLE 5:** The top 10 KGRDR-predicted candidate drugs for AD.

Rank	Candidate drugs	PubMed Id
1	Escitalopram	3,2,741,828
2	Tamoxifen	33,831,349
3	Tacrolimus	17,270,732
4	Amitriptyline	21,738,757
5	Phenylbutyric acid	37,354,655
6	Rifampin	22,718,435
7	Mechlorethamine	7,885,382
8	Prochlorperazine	17,047,137
9	Hyoscyamine	36,508,538
10	Chlorpromazine	27,458,372

For PD, we focused on analyzing the top 10 candidate drugs predicted by KGRDR. As shown in Table 6, we found that 9 out of 10 drugs (90% success rate) have been verified by the reliable sources and clinical trials. For example, KGRDR predicted that Biperiden affects both the central and peripheral nervous systems. It has been approved for the treatment of arteriosclerosis, idiopathic and post-encephalitic Parkinson’s syndrome. This drug-disease association has also been recorded in DrugBank (Wishart et al., 2018). Therefore, Biperiden is the first potential drug predicted in this article to treat Parkinson’s disease. In addition, Orphenadrine predicted by KGRDR as an adjuvant drug for relieving musculoskeletal pain and discomfort symptoms has also been shown to be useful for the treatment of drug-induced Parkinson’s syndrome and the relief of pain caused by muscle spasms. This prediction is also supported by DrugBank and PubChem.

**TABLE 6:** The top 10 KGRDR-predicted candidate drugs for PD.

Rank	Candidate drugs	PubMed Id
1	Biperiden	31,643,581
2	Orphenadrine	3,741,741
3	Oxprenolol	4,114,766
4	Trospium	36,625,617
5	Betaine	36,735,640
6	Hydromorphone	NA
7	Methadone	27,871,509
8	Ziconotide	26,362,469
9	Streptomycin	38,174,534
10	Goserelin	19,222,889

To sum up, most of our predictions can be verified by reliable sources and clinical trials. The case study results further demonstrate the effectiveness of KGRDR in predicting novel drug–disease associations.

## 4 Conclusion

In this study, we proposed a new framework for drug-disease interaction prediction by combining graph regularized integration and knowledge graph embedding, named KGRDR. Firstly, in order to alleviate the data quality problem caused by using single feature information of drugs and diseases, the graph regularized integration method is applied to fuse the drug and disease similarities from multiple data sources to build a more comprehensive heterogeneous network with multiple relationship types to improve the prediction performance of the model. Secondly, most of the current studies on drug-disease interaction prediction only consider drug and disease entities, ignoring the association between drugs or diseases and other entities during the drug’s efficacy. Therefore, we introduce the knowledge graph embedding module to obtain the global topological feature representation of drug and disease entities by constructing the association between drugs and diseases and multiple medical entities. Finally, the attention feature fusion method is used to fuse the feature information of the graph regularized integration module and the knowledge graph embedding module, and feed the fused features into the graph convolutional network prediction model to identify the potential therapeutic indications of drugs. The results of extensive experiments demonstrated that KGRDR outperformed other drug–disease association prediction methods and various variants of KGRDR.

In summary, KGRDR integrates multiple similarity information between drugs and diseases, and obtains various topological associations in drug-disease heterogeneous networks based on knowledge graphs, which can significantly improve the performance of drug-disease interaction prediction models. It can help pharmacologists or biologists effectively narrow down the search space of candidate drugs. It may further guide them to conduct wet-lab experiments and thus reduce costs and time. In future work, more biomedical information can be integrated to learn drug and disease features, and efficient fusion strategies can be designed to obtain more comprehensive feature information.

## Data Availability

The original contributions presented in the study are included in the article/supplementary material, further inquiries can be directed to the corresponding author.
